# Supplementary material for: Comparative multiomics analysis of cell physiological state after culture in a basket bioreactor
Source: Sci Rep. 2022 Nov 23;12:20161. doi: 10.1038/s41598-022-24687-4 (PMC9686226; doi:10.1038/s41598-022-24687-4)
Supplement: Supplementary file 1 — Supplementary Information 1. [file 41598_2022_24687_MOESM1_ESM.zip › raw data/Proteomic raw data/6.KEGGenrichment/EG.vs.CG/ALL/pathway/src/00910.html]

map00910


- K01672, K18245, K18246, K01673, K01674, 4.2.1.1, R10092"

- Down regulated proteins

A0A0D9RIT7(Description:Carbonic anhydrase;Gene:CA12;log2FC:-1.6428)

A0A0D9RQX6(Description:Carbonic anhydrase;Gene:CA5B;log2FC:-inf)

Close
